# Development of a nomogram for predicting anastomotic leakage after rectal cancer surgery incorporating inflammatory indices

**DOI:** 10.3389/fonc.2026.1806208

**Published:** 2026-05-21

**Authors:** Feihong Zhao, Dongjie Zheng, Weiqiang Zhang, Yongzeng Liu, Tie Wang

**Affiliations:** Department of General Surgery, CangZhou Hospital of Integrated Traditional Chinese and Western Medicine in Hebei Province, Cangzhou, China

**Keywords:** anastomotic leakage, nomogram, rectal cancer, risk prediction, systemic inflammation response index (SIRI)

## Abstract

**Objective:**

Anastomotic leakage (AL) is a common and serious complication after rectal cancer surgery, and there is still a lack of effective prediction tools. This study aimed to build a prediction model for AL after rectal cancer surgery by combining inflammatory indicators.

**Methods:**

We collected clinical data from 650 patients who underwent anterior resection for rectal cancer in the General Surgery Department of Cang Zhou Hospital of Integrated Traditional Chinese and Western Medicine of Hebei Province between January 2020 and December 2024. The Least Absolute Shrinkage and Selection Operator (LASSO) regression was used to screen predictors. A multivariate logistic regression was then used to build the prediction model, presented as a nomogram. The model’s performance was evaluated using the Receiver Operating Characteristic (ROC) curve, Calibration curves and Decision Curve Analysis (DCA) further demonstrated acceptable calibration and suggested potential clinical utility.

**Results:**

Data from 650 rectal cancer patients were included, with an average age of 63.06 ± 9.84 years. Multivariate logistic regression identified six independent predictors for AL: male gender (OR=1.675, 95% CI: 1.020-2.755), low tumor location (tumor location<7cm, OR=1.795, 95% CI: 1.126-2.862), high BMI (OR=2.176, 95% CI: 1.607-2.948), longer operation time (OR=2.697, 95% CI: 1.942-3.745), blood loss (OR=0.520, 95% CI: 0.403-0.671), and high Systemic Inflammation Response Index (SIRI) (OR=1.520, 95% CI: 1.278-1.808). The nomogram model demonstrated good predictive performance in both the training and validation sets, with Area Under the Curve (AUC) values of 0.763 (95% CI: 0.706-0.820) and 0.798 (95% CI: 0.707-0.889), respectively. Calibration curves and Decision Curve Analysis (DCA) indicated acceptable calibration and potential clinical utility.

**Conclusion:**

This study developed and validated a nomogram prediction model that combines preoperative inflammatory indicators (SIRI) with key clinical features. This model can help clinicians identify high-risk patients early and provides a quantitative tool for implementing targeted perioperative intervention strategies.

## 1 Introduction

Rectal cancer is a common gastrointestinal malignancy worldwide. Its incidence is among the highest for cancers, and it has shown a yearly increasing trend in recent years. It has high mortality and poor prognosis (1). Currently, surgical resection remains the core treatment for resectable rectal cancer. Anterior resection for rectal cancer is one of the preferred surgical methods because it can best preserve anal function and improve postoperative quality of life (2). However, postoperative complications remain a major challenge for surgeons. AL is one of the most serious complications. Its early detection and treatment are still key and difficult research areas in rectal cancer surgery. Even though surgery has improved greatly over the past decades, the actual incidence of AL has hardly decreased. Reported rates are roughly between 10% and 20% (3, 4). Also, AL is linked to various poor outcomes. Studies show it increases local recurrence rates and is associated with worse long-term survival (5, 6). Therefore, it is crucial to early identify and predict individuals at high risk for postoperative leakage and to develop effective prevention and management strategies.

In recent years, several studies have explored risk factors for AL after colorectal cancer surgery. Based on this, prediction models or risk scores have been developed (7–9). These models or scoring systems are mainly based on patient baseline characteristics or tumor clinical features. They provide important basis for clinical risk stratification. However, existing models or scores do not systematically integrate preoperative hematological indicators, especially for low rectal cancer. Some studies have noted the potential impact of preoperative inflammation-related indicators (10, 11). But composite indicators like the Systemic Immune-Inflammation Index (SII) and Systemic Inflammation Response Index (SIRI) have not been fully explored. So, some potential and important influencing factors may be missed. In fact, preoperative inflammation and nutrition-related indicators may reflect the body’s underlying inflammatory state, tumor biology, and nutritional status. These factors are closely related to anastomotic healing.

Currently, there is a lack of research combining preoperative inflammation-related indicators to predict AL after rectal cancer surgery. Therefore, this study retrospectively analyzed clinical data from 650 patients who underwent anterior resection for rectal cancer at our hospital from January 2020 to December 2024. It aimed to explore the impact of preoperative hematological indicators on AL. It also aimed to build a prediction model combining inflammation-related indicators. This could provide a basis for achieving “precise prevention and early intervention” in clinical practice, thereby helping to reduce both the incidence of AL and its associated risks.

## 2 Materials and methods

### 2.1 Patient selection

**Figure 1:**
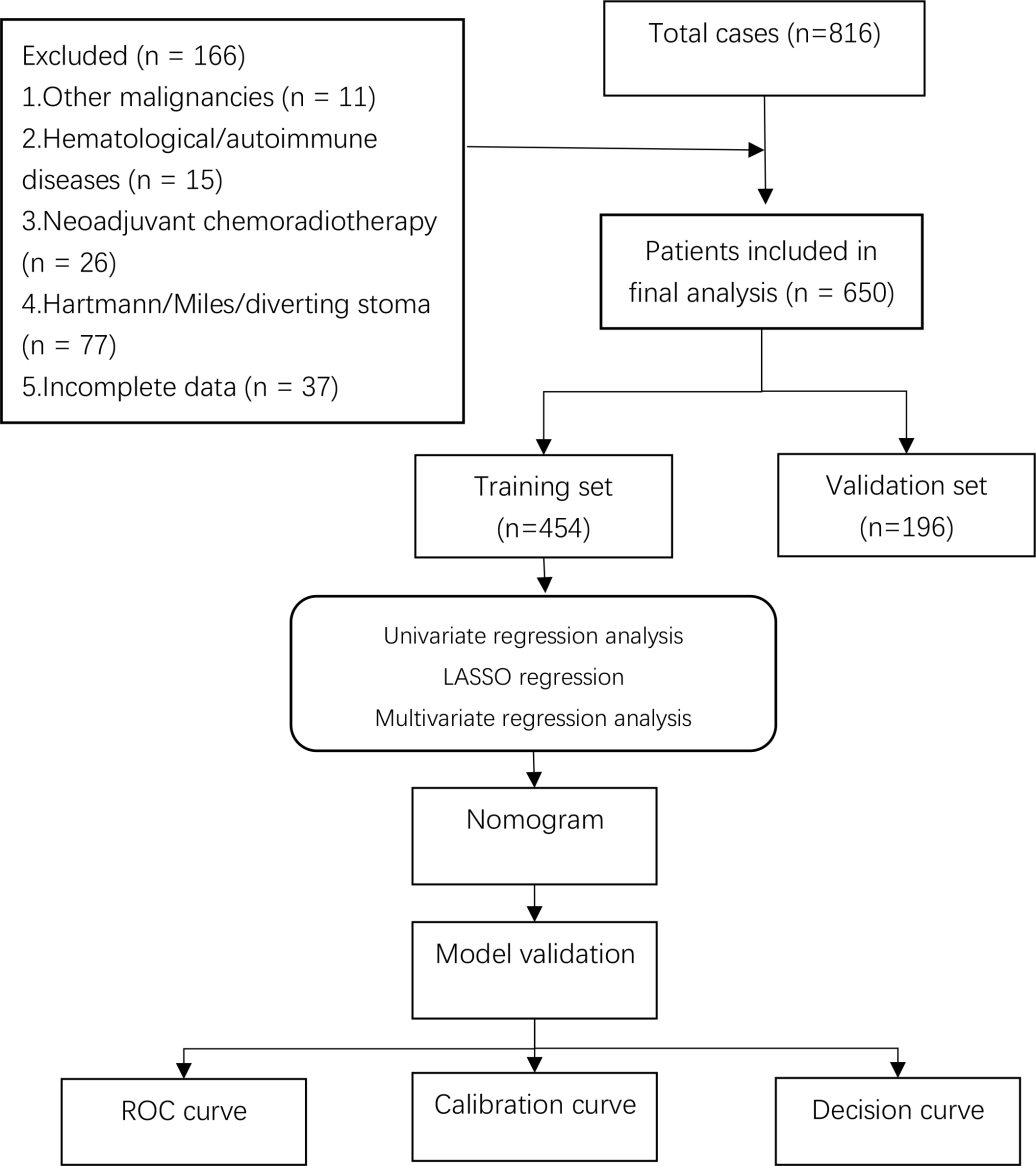
Flow chart.

This study included clinicopathological data from 650 patients who underwent laparoscopic anterior resection for rectal cancer at Cang Zhou Hospital of Integrated Traditional Chinese and Western Medicine of Hebei Province between January 2020 and December 2024. The flow chart is shown in **Figure 1**.

Inclusion criteria: (1) Complete case data without missing information; (2) Pathologically confirmed rectal cancer; (3) Primary laparoscopic anterior resection performed.

Exclusion criteria: (1) Combined with other malignant tumors; (2) preoperative hematological diseases or autoimmune diseases; (3) patients who received preoperative neoadjuvant chemoradiotherapy; (4) patients who underwent Hartmann or Miles procedures.

This study was approved by the Ethics Committee of Cang Zhou Hospital of Integrated Traditional Chinese and Western Medicine of Hebei Province.

### 2.2 Definition of anastomotic leakage

The International Rectal Cancer Research Group defined postoperative AL in 2010 as a disruption or defect in the intestinal wall at the anastomosis site, creating communication between the intestinal lumen and the peritoneal cavity. Specific grades are: Grade A is subclinical or radiological AL, usually without symptoms and needing no special treatment. Grade B AL shows clinical symptoms like abdominal pain, fever, purulent or fecal discharge from drain or vagina, usually requiring conservative treatment. Grade C AL shows symptoms like peritonitis or sepsis, requiring re-operation. The outcome diagnosis in this study strictly followed this definition, and all cases of AL (Grades A, B, and C) were included.

### 2.3 Data collection

Patient basic information, pathological data, and laboratory test results were collected from the hospital electronic database. Basic information included gender, age, body mass index (BMI), operation time, blood loss, etc. Pathological data included tumor location, stage, longest diameter, differentiation degree, depth of invasion, metastasis, etc. Laboratory tests were results from within one week before surgery. They included neutrophil count, lymphocyte count, monocyte count, carcinoembryonic antigen (CEA), carbohydrate antigen 19-9 (CA19-9), platelet count (Plt), prothrombin time (PT), activated partial thromboplastin time (APTT), thrombin time (TT), fibrinogen (Fib). The following ratios were calculated: Neutrophil-to-lymphocyte ratio (NLR): neutrophil count ÷ lymphocyte count; Platelet-to-lymphocyte ratio (PLR): platelet count ÷ lymphocyte count; Monocyte-to-lymphocyte ratio (MLR): monocyte count ÷ lymphocyte count; Platelet-lymphocyte-monocyte index (PIV): platelet count × monocyte count ÷ lymphocyte count; Systemic Immune-Inflammation Index (SII): platelet count × neutrophil count ÷ lymphocyte count; Systemic Inflammation Response Index (SIRI): neutrophil count × monocyte count ÷ lymphocyte count; Prognostic Nutritional Index (PNI): serum albumin (g/L) + 5 × lymphocyte count (×10^9^/L); Albumin-to-globulin ratio (AGR): serum albumin (g/L) ÷ serum globulin (g/L).

### 2.4 Statistical analysis

Statistical analysis was performed using R V4.2.3 and SPSS 26.0 software. Measurement data conforming to normal distribution are expressed as mean ± standard deviation ( $\bar{x}$ ± s), and compared between groups using independent samples t-test. Categorical data are expressed as number or percentage, and compared using χ² test or Fisher’s exact test. Patients were randomly divided into a training set (n=454) and a validation set (n=196) in a 7:3 ratio. In the training set, univariate logistic regression analysis was performed (P<0.05 as potential predictors), and multicollinearity was tested (VIF<10). Continuous variables, including BMI, operation time, blood loss, and SIRI, were retained as continuous values without categorization. Tumor location was dichotomized using a predefined clinical threshold (<7cm vs ≥7 cm). No data-driven cut-offs were applied. LASSO regression was then used to further screen variables, followed by multivariate logistic regression to identify independent predictors and build the nomogram model. The model’s discrimination, calibration, and clinical usefulness were evaluated using ROC curve, calibration curve (Bootstrap 1000 times), and Decision Curve Analysis (DCA). Internal validation was performed in the validation set.

## 3 Results

### 3.1 Basic patient characteristics

This study included 650 patients. Among them, 135 (20.8%) developed AL. The demographic and clinical characteristics were well-balanced between the training and validation sets (all P > 0.05). The overall average age was 63.06 ± 9.84 years. There were 418 males (64.3%) and 232 females (35.7%). Compared with the no-leakage group, the AL group had significantly higher rates of hypertension, higher BMI, longer operation time, more low tumors (tumor location ≤ 7cm), larger tumor diameter, higher N stage, and higher levels of several inflammation-related indicators (including MLR, PIV, SII, and SIRI) (P< 0.05) (**Tables 1**, **2**).

**Table 1:** Comparison of baseline characteristics between the two groups.

Variable	No leak (n=515)	Leak (n=135)	p
Gender			0.051
Male	321 (62)	97 (72)	
Female	194 (38)	38 (28)	
Age	63.26 ± 10.01	62.15 ± 9.45	0.207
Diabetes			0.506
No	436 (85)	118 (87)	
Yes	79 (15)	17 (13)	
Hypertension			0.011
No	380 (74)	84 (62)	
Yes	135 (26)	51 (38)	
BMI	23.1 ± 2.44	24.33 ± 2.27	
Tumor Location			0.002
>7cm	253 (49)	46 (34)	
≤7cm	262 (51)	89 (66)	
Tumor Diameter	4.53 ± 1.43	4.81 ± 0.89	0.005
Tumor Differentiation			0.687
Low	19 (4)	7 (5)	
Moderate	392 (76)	103 (76)	
High	104 (20)	25 (19)	
T			0.482
1-2	76 (15)	22 (16)	
3-4	439 (85)	113 (84)	
N			0.043
0-1	410 (80)	117 (87)	
2	105 (20)	18 (13)	
Tumor Stage			0.103
I-II	258 (50)	81 (60)	
III	252 (49)	53 (39)	
IV	5 (1)	1 (1)	
Operation Time	131.92 ± 35.32	150.79 ± 34.18	< 0.001
Blood loss	111.70 ± 58.95	99.32 ± 72.99	0.011

**Table 2:** Comparison of laboratory indicators between the two groups.

Variable	No leak	Leak	p
WBC	6.67 ± 1.97	7.21 ± 1.93	0.004
RBC	4.49 ± 0.56	4.64 ± 0.45	0.006
Hemoglobin	135.92 ± 20.06	130.36 ± 17.26	0.135
Platelet	253.82 ± 63.42	258.54 ± 59.67	0.164
CEA	13.27 ± 22.49	18.93 ± 30.07	0.087
CA-199	22.27 ± 40.28	24.52 ± 36.46	0.81
PT	11.37 ± 1.38	11.45 ± 1.49	0.523
APTT	33.81 ± 3.08	34.22 ± 3.65	0.27
TT	13.63 ± 1.43	13.32 ± 0.73	0.007
Fib	3.15 ± 0.57	3.28 ± 0.85	0.196
NLR	2.53 ± 1.04	2.82 ± 1.50	0.086
PLR	152.53 ± 68.34	143.74 ± 31.99	0.393
MLR	0.18 ± 0.09	0.21 ± 0.14	0.039
PIV	206.52 ± 163.56	305.73 ± 293.67	< 0.001
SII	656.02 ± 383.13	739.93 ± 351.22	0.033
SIRI	0.79 ± 0.48	1.07 ± 0.97	0.004
PNI	51.72 ± 5.29	51.11 ± 3.81	0.189
AGR	1.51 ± 0.27	1.46 ± 0.30	0.137

### 3.2 Univariate analysis and multicollinearity analysis for postoperative AL

**Figure 2:**
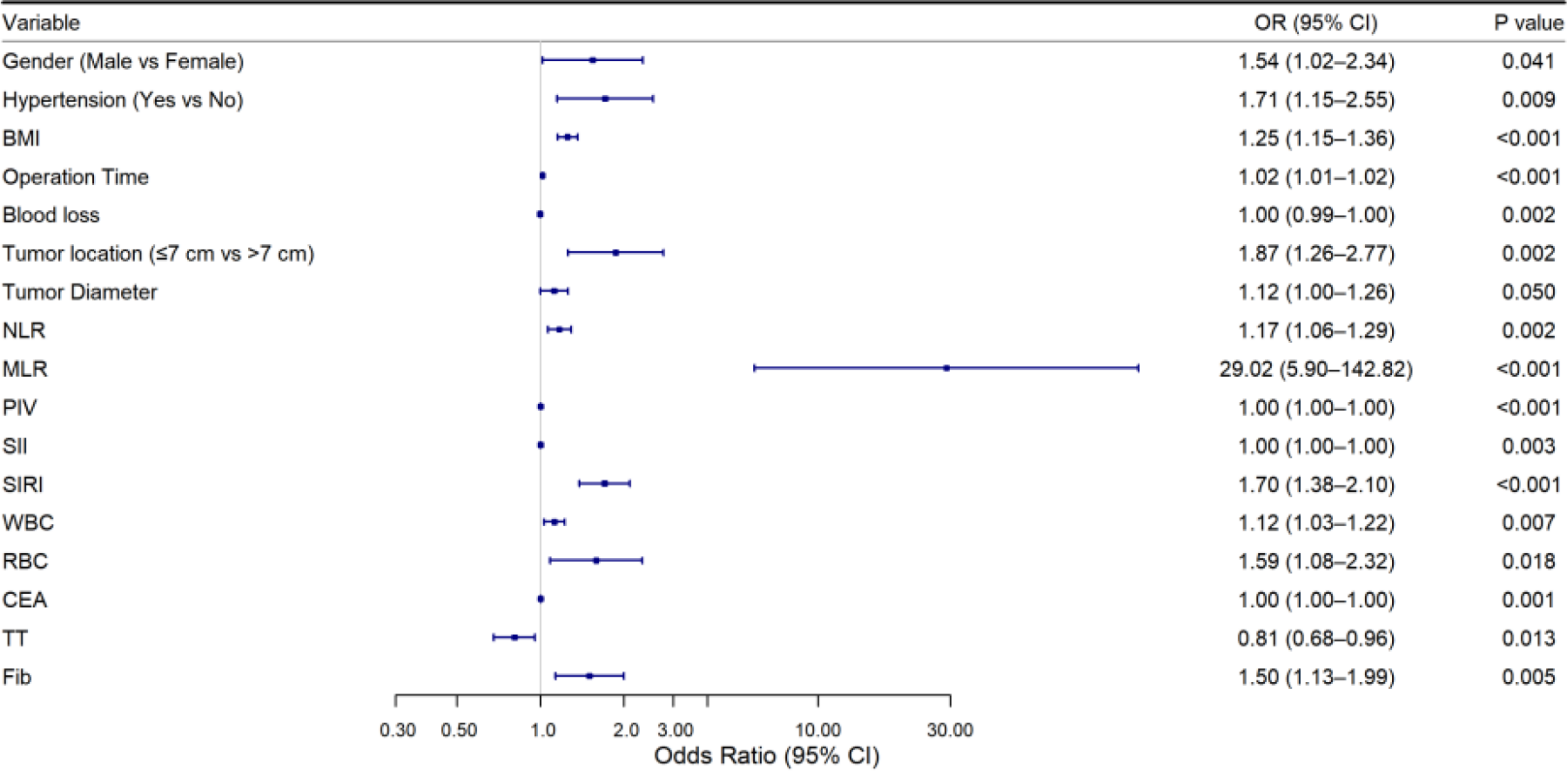
Forest plot of univariable logistic regression analysis.

**Figure 3:**
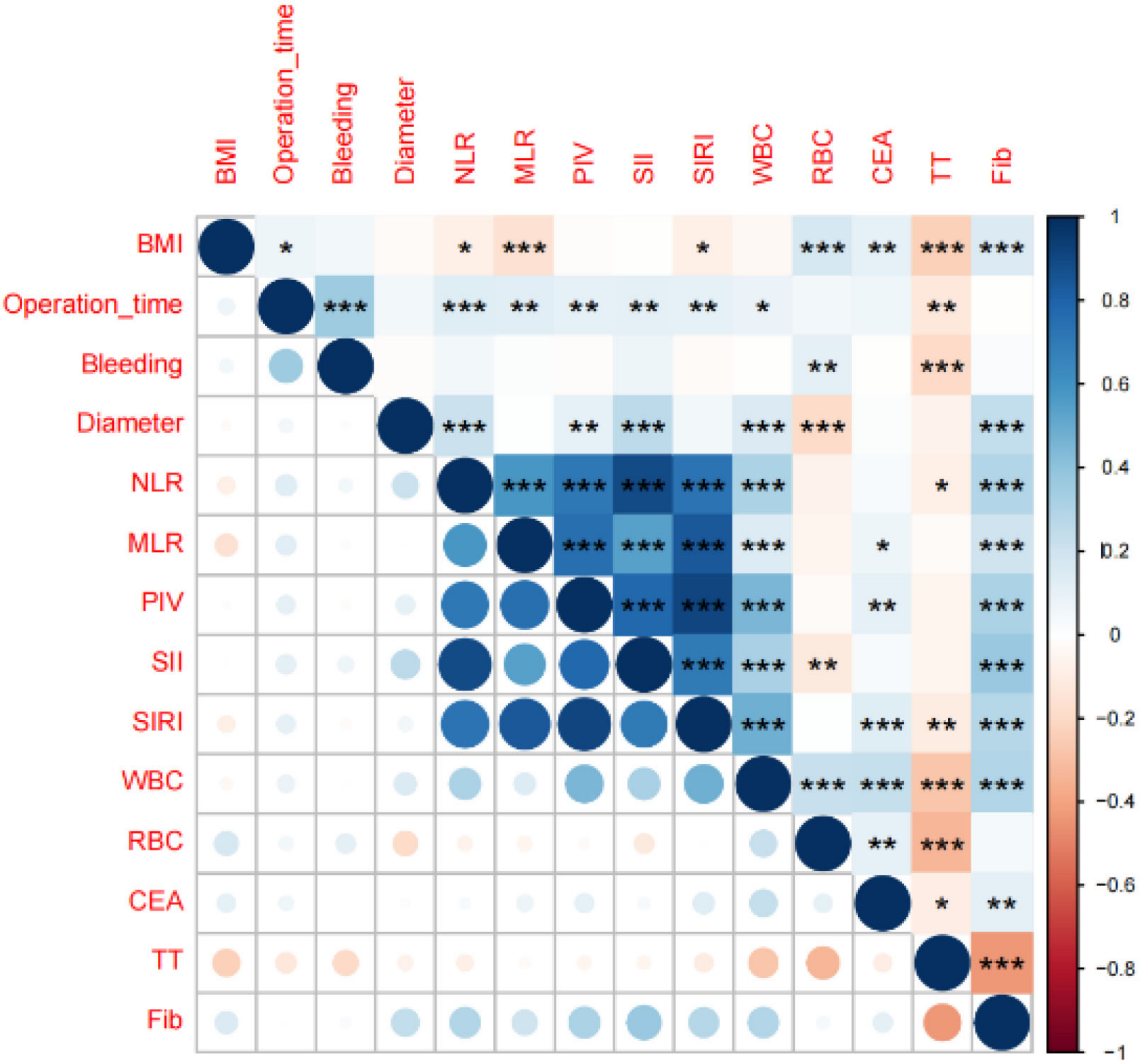
Multicollinearity analysis. *p < 0.05, **p < 0.01, ***p < 0.001.

In the training set, univariate logistic analysis showed that male gender, hypertension, high BMI, longer operation time, lower blood loss, low tumor location, and larger tumor diameter were risk factors for postoperative AL. Among inflammation-related indicators, higher NLR, MLR, PIV, SII, SIRI, WBC, RBC, CEA, and Fib increased the risk of postoperative AL. Higher TT decreased the risk (**Table 3**, **Figure 2**). Also, multicollinearity analysis was performed on these potential risk factors. The results showed the Variance Inflation Factor (VIF) for each variable was<10. This indicated no significant multicollinearity, so they could be included in further analysis (**Figure 3**).

**Table 3:** Univariate logistic regression analysis.

Variable	B value	SE	OR (95%CI)	Wald	P
Gender (Female=0; Male=1)	0.434	0.212	1.543(1.018-2.337)	2.046	0.041
Hypertension (No=0; Yes=1)	0.536	0.204	1.709(1.146- 2.548)	2.629	0.009
BMI	0.224	0.043	1.251(1.151-1.361)	5.248	<0.001
Operation Time	0.016	0.003	1.016(1.011-1.022)	5.646	<0.001
Blood loss	-0.004	0.001	0.996(0.994-0.999)	3.047	0.002
Tumor Location (≥7 = 0; <7 = 1)	0.625	0.202	1.868(1.258-2.775)	3.097	0.002
Tumor Diameter	0.114	0.058	1.121(1.000-1.257)	1.962	0.050
NLR	0.157	0.049	1.170(1.062-1.289)	3.171	0.002
MLR	3.368	0.813	29.019(5.896-142.822)	4.142	<0.001
PIV	0.001	0.000	1.001(1.001-1.002)	4.317	<0.001
SII	0.000	0.000	1.000(1.000-1.001)	2.989	0.003
SIRI	0.532	0.107	1.703(1.381-2.100)	4.983	<0.001
WBC	0.117	0.043	1.124(1.033-1.224)	2.701	0.007
RBC	0.461	0.195	1.586(1.083-2.323)	2.371	0.018
CEA	0.001	0.000	1.001(1.001-1.002)	3.466	0.001
TT	-0.216	0.087	0.806(0.679-0.956)	2.476	0.013
Fib	0.406	0.145	1.502(1.131-1.994)	2.810	0.005

### 3.3 LASSO regression cross-validation and multivariate logistic regression analysis

**Figure 4:**
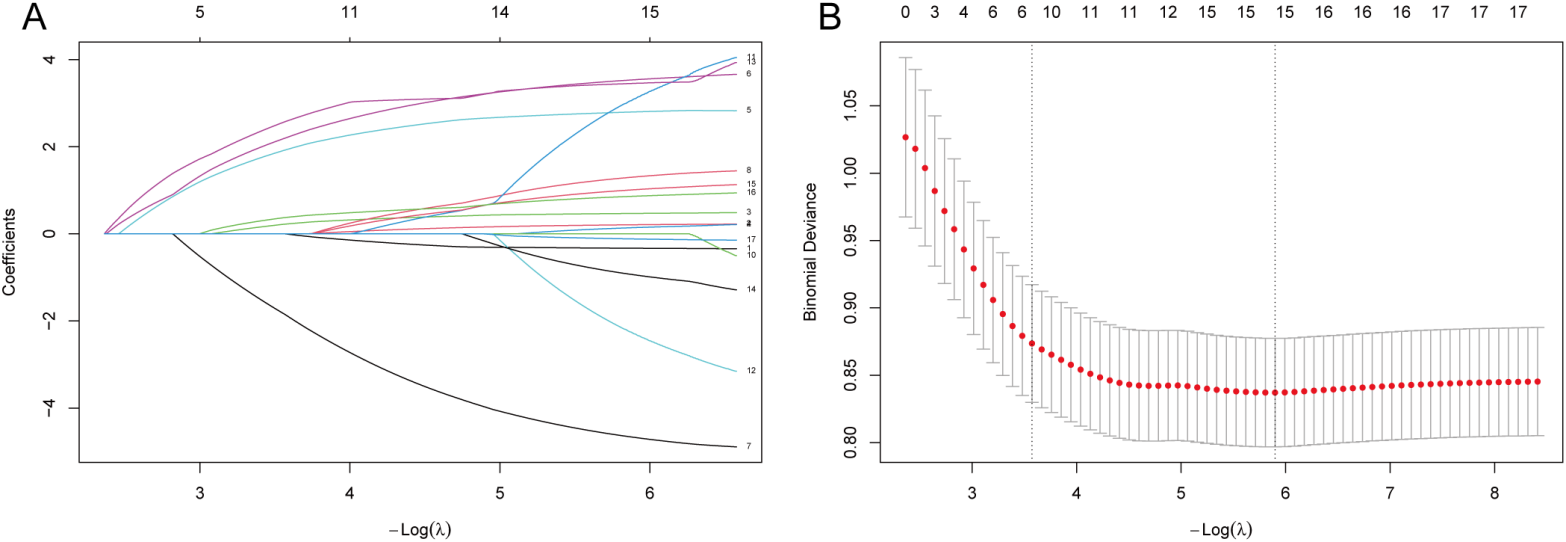
LASSO regression variable selection. **(A)** Coefficient paths for each predictor. Variables were retained at lambda.1se **(B)** Ten-fold cross-validation plot. The left vertical dashed line represents lambda.1se (the selected optimal value), and the right vertical dashed line represents lambda.min. The number of predictors corresponding to each lambda is shown above.

**Figure 5:**
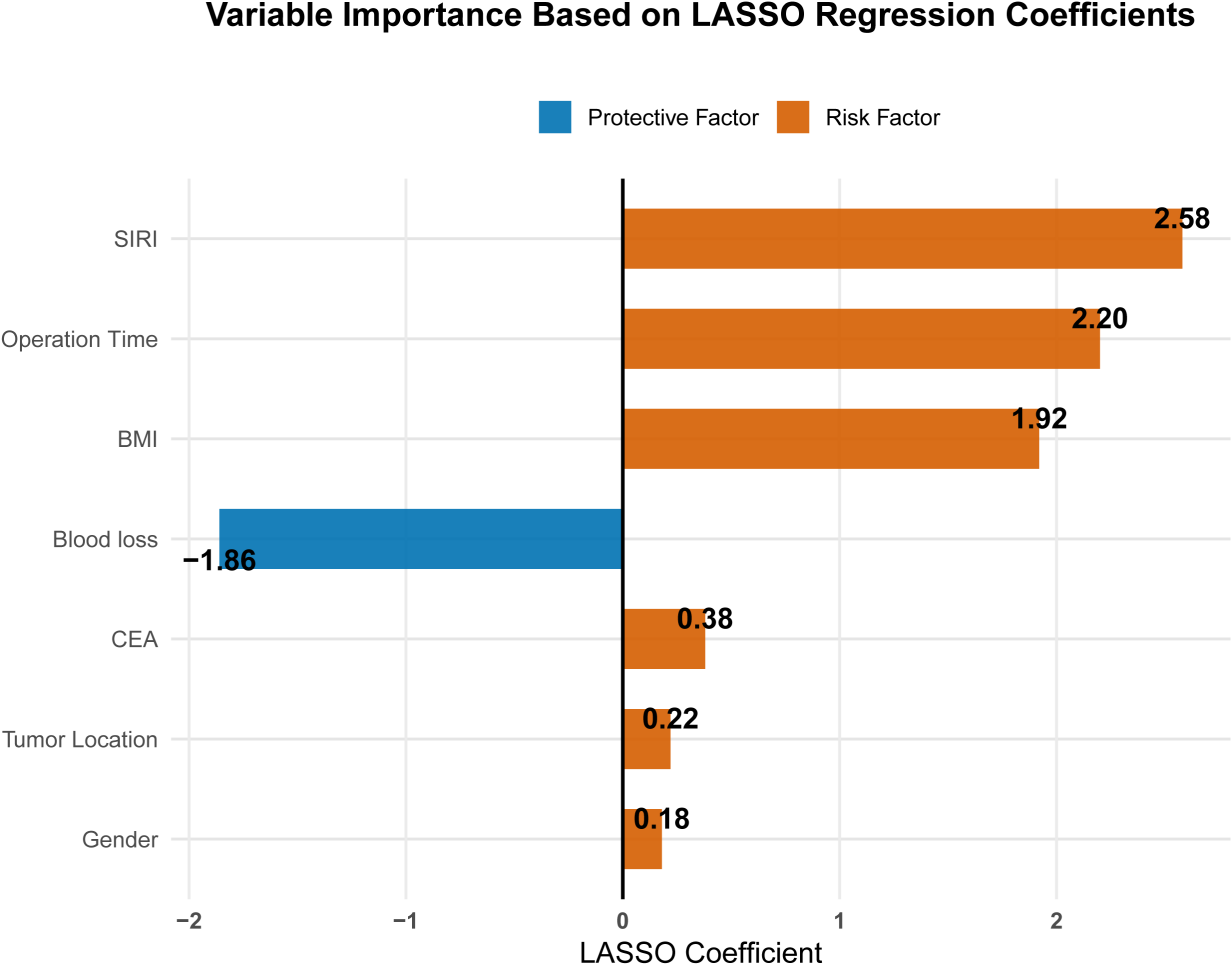
Variable importance plot based on LASSO regression coefficients.

LASSO regression was used to screen potential predictors from the 17 variables significant in univariate analysis. Based on the optimal penalty parameter selected by ten-fold cross-validation (lambda.1se), seven variables with non-zero coefficients were retained: gender, tumor location, BMI, operation time, blood loss, SIRI, and CEA (**Figure 4**). The corresponding LASSO regression coefficients for these predictors are presented in **Table 4**, and the relative importance of each variable (based on absolute coefficient magnitude) is visualized in **Figure 5**.

**Table 4:** LASSO regression coefficients of predictors selected at lambda.1se.

Variable	LASSO Coefficient
Gender	0.18
BMI	1.92
Operation Time	2.20
Blood loss	-1.86
SIRI	2.58
CEA	0.38
Tumor Location	0.22

**Figure 6:**
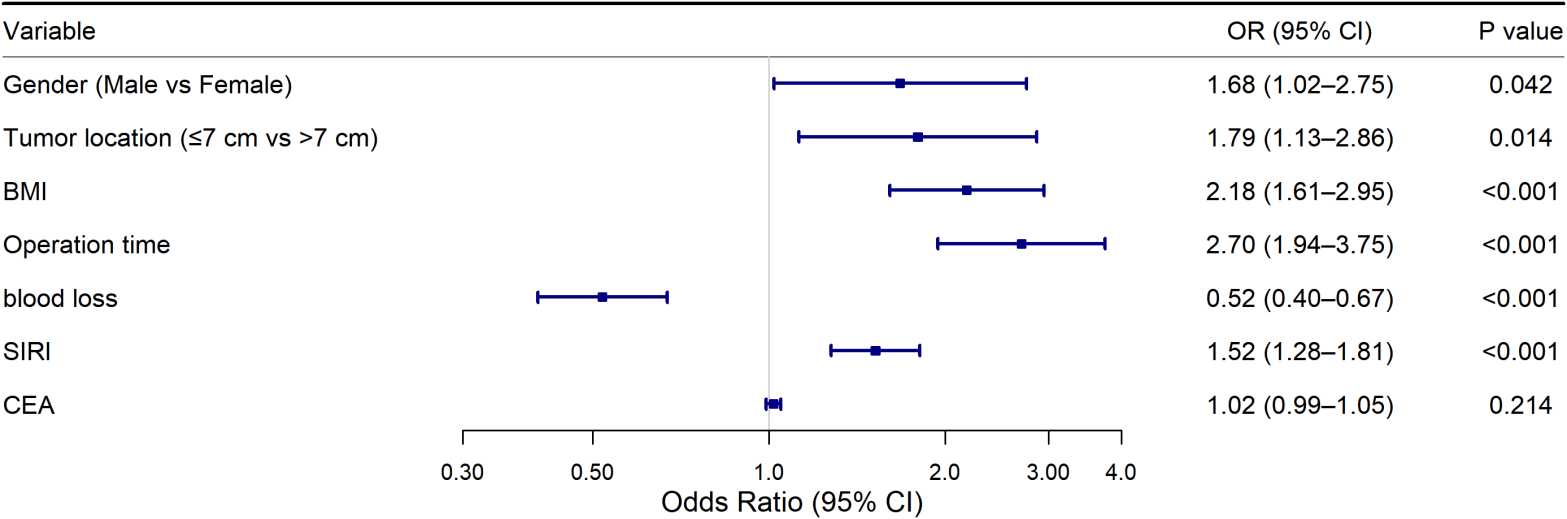
Forest plot of multivariable logistic regression analysis.

These seven variables were then included in the multivariate logistic regression model. The results showed that CEA was not statistically significant (OR=1.21, 95% CI: 0.89–1.64, P=0.214). Therefore, the final nomogram model was built using the remaining six independent predictors (**Table 5**, **Figure 6**).

**Table 5:** Multivariate logistic regression analysis.

Variable	B value	SE	OR (95%CI)	Wald	P
Gender (Female=0; Male=1)	0.517	0.253	1.675(1.020-2.755)	4.13	0.042
Tumor Location (≥7 = 0; <7 = 1)	0.585	0.238	1.795(1.126-2.862)	6.01	0.0142
BMI	0.778	0.155	2.176(1.607-2.948)	25.13	<0.001
Operation Time	0.992	0.168	2.697(1.942-3.745)	35.56	<0.001
Blood loss	-0.654	0.130	0.520(0.403-0.671)	25.60	<0.001
SIRI	0.419	0.089	1.520(1.278-1.808)	22.34	<0.001
CEA	0.018	0.015	1.018(0.990-1.048)	1.54	0.214

### 3.4 Establishment of the nomogram model

**Figure 7:**
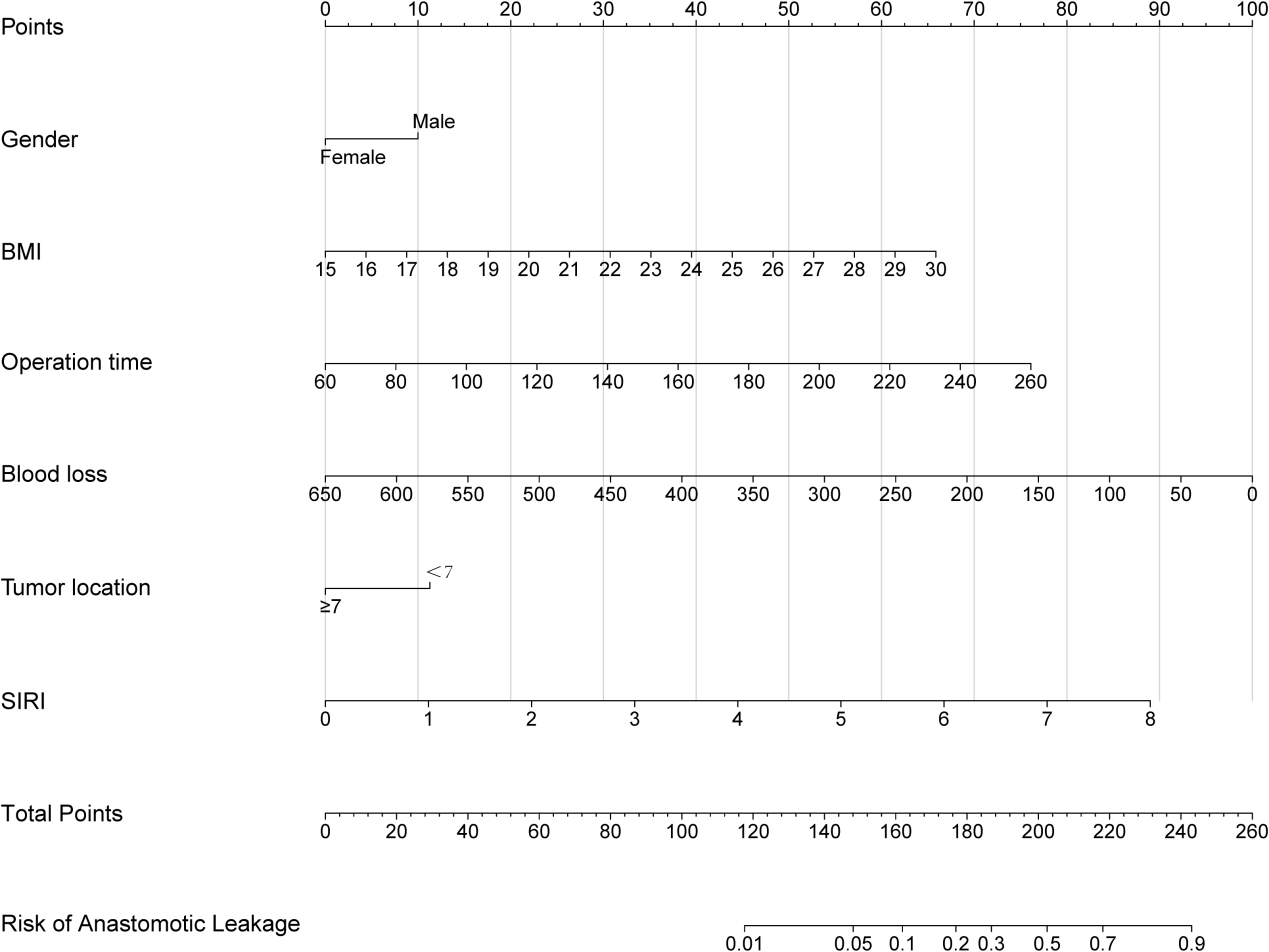
Nomogram prediction model for AL after rectal cancer surgery.

A nomogram prediction model was built based on the above 6 variables (**Figure 7**). Each variable was assigned a score according to its contribution. The total score corresponds to the probability of AL occurrence.

### 3.5 Evaluation and validation of the nomogram model

**Figure 8:**
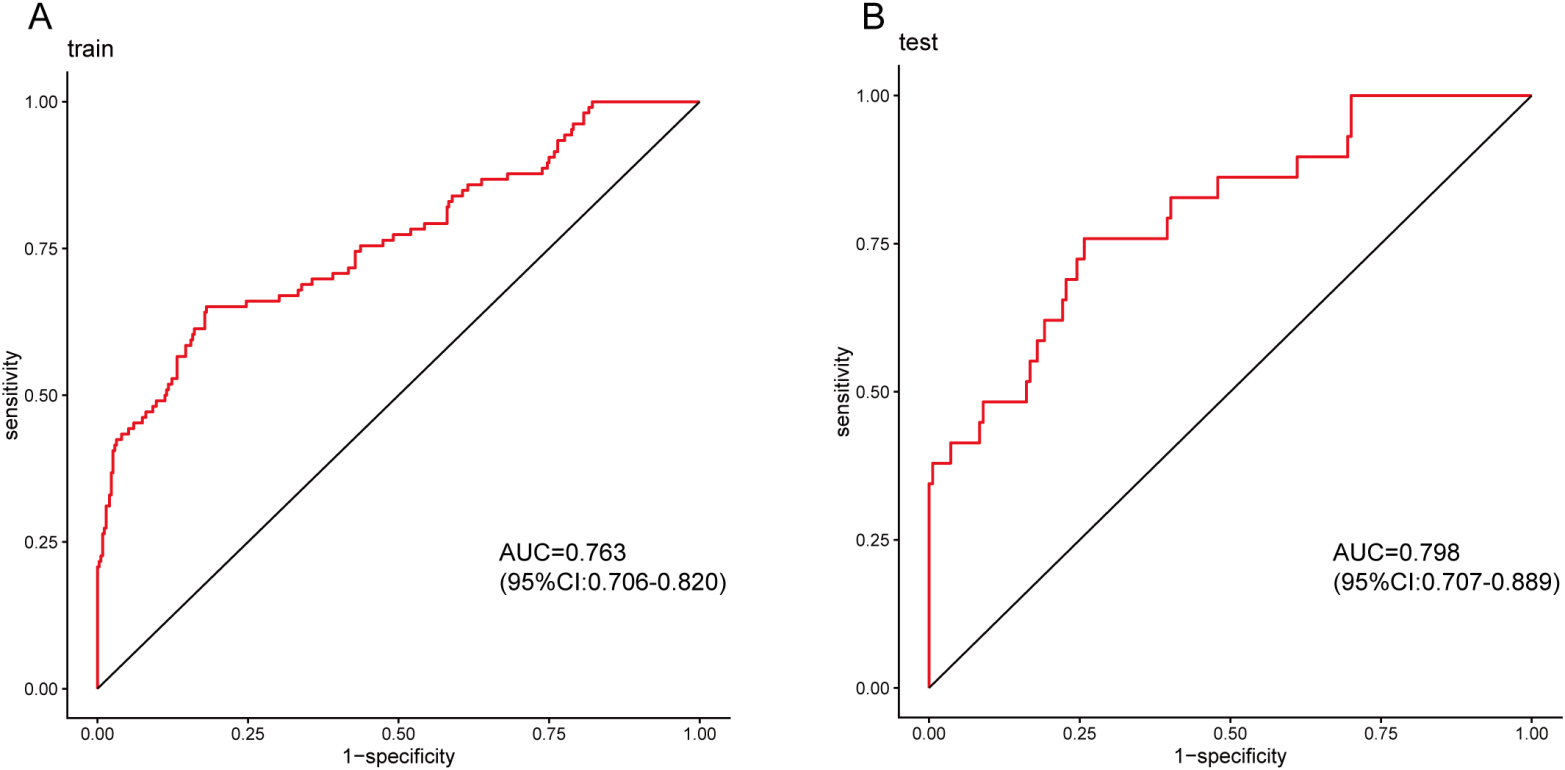
ROC curves of the model in training and validation sets. **(A)** ROC curve for the training set; **(B)** ROC curve for the validation set.

**Figure 9:**
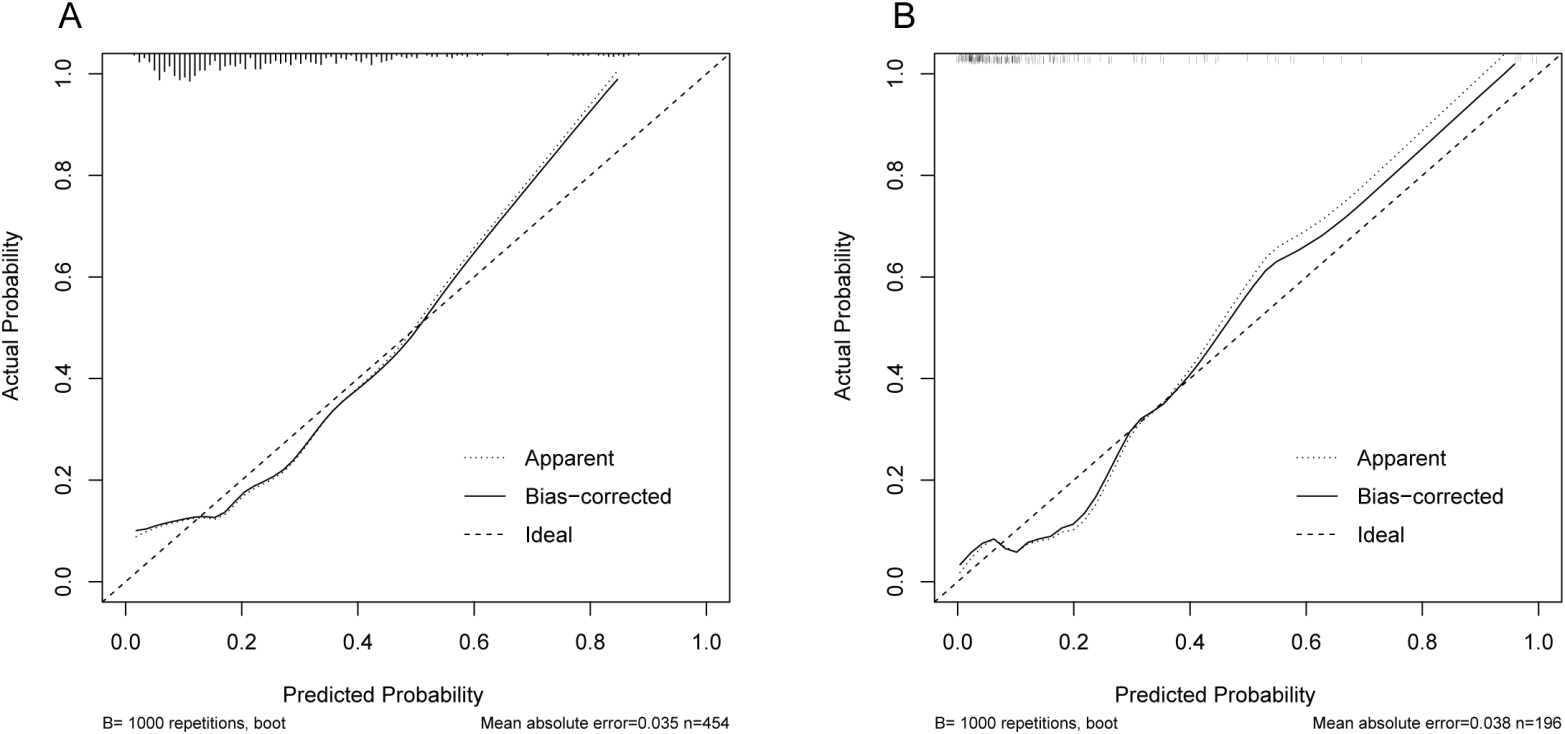
Calibration curves of the model in training and validation sets. **(A)** Calibration curve for the training set; **(B)** Calibration curve for the validation set.

**Figure 10:**
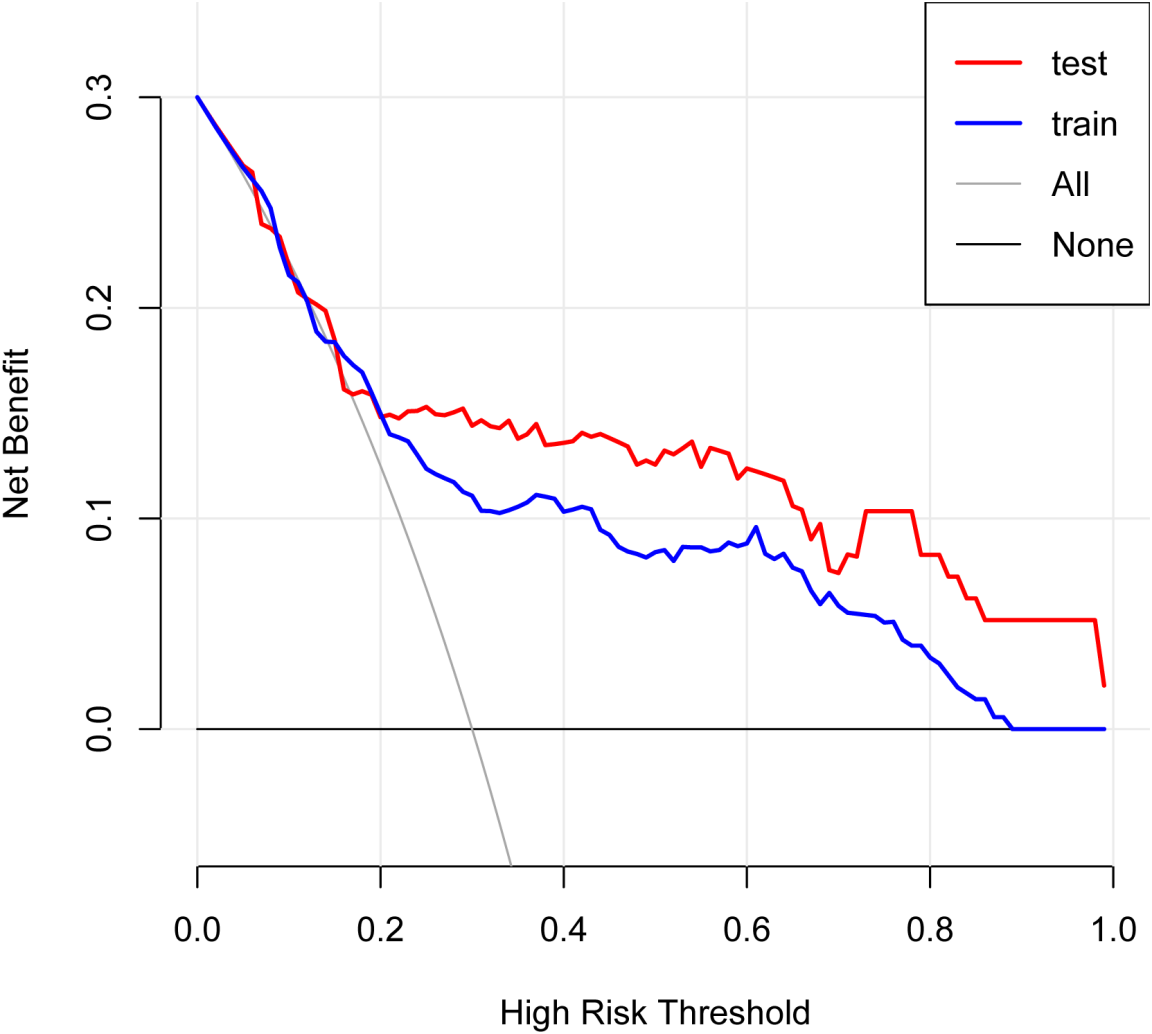
Decision curves of the model in training and validation sets.

The model performed well in both the training and validation sets. In the training set, AUC=0.763 (95% CI: 0.706-0.820). In the validation set, AUC=0.798 (95% CI: 0.707-0.889), see **Figure 8**. After 1000 Bootstrap resampling corrections, the calibration curves for both training and validation sets were close to the ideal diagonal line (**Figure 9**). Quantitative assessment further supported good calibration, with Brier scores of 0.125 and 0.126 for the training and validation sets, respectively, and a calibration slope of 1.077 in the validation set, indicating no substantial overfitting. Clinical usefulness was shown by Decision Curve Analysis (DCA). Over a wide range of threshold probabilities, this model provided higher net benefit compared to “treat all” or “treat none” strategies (**Figure 10**). This confirms its good clinical application value.

## 4 Discussion

This study analyzed 650 patients who underwent anterior resection for rectal cancer and systematically evaluated the predictive value of various inflammation and nutrition-related indicators (NLR, PLR, MLR, PIV, SII, SIRI, PNI, AGR) for anastomotic leakage. A nomogram incorporating preoperative inflammatory markers and clinical features was developed and internally validated. The model demonstrated good discriminatory capacity, with AUC values of 0.763 in the training cohort and 0.798 in the validation cohort. Although these values reflect acceptable predictive performance, they imply a non-negligible rate of misclassifications that warrants consideration in clinical application. Compared with previously published models for predicting anastomotic leakage after rectal cancer surgery our model performs comparably or slightly better (12), with the distinct advantage of including SIRI, which may capture perioperative systemic inflammation more comprehensively. Notably, among the various inflammatory indicators, SIRI emerged as an independent risk factor, integrating a quantifiable measure of systemic inflammation into perioperative risk assessment and offering a new perspective for individualized management. Multivariable analysis identified six independent predictors: male sex, low tumor location (<7cm from the anal verge), high BMI, longer operation time, lower blood loss, and elevated SIRI.

Many previous studies have confirmed that male gender is a risk factor for AL after rectal cancer surgery. This is consistent with our findings. Sparreboom et al. (13) determined through multivariate logistic regression that male gender is an independent risk factor for AL. Another study (14) also showed a clear association between male gender and AL. The reason may be that the male pelvis is usually narrower than the female pelvis. This makes surgery more complex. To fully expose the surgical field, it may increase damage to adjacent tissues and affect blood supply to the anastomosis, raising the risk of postoperative AL. In contrast, females may avoid these injuries due to a wider pelvis, thus lowering the risk (15–17). Also, androgens in males may somewhat inhibit inflammatory response and tissue repair, leading to relatively weaker anastomotic healing ability (18).

Low tumor location is also an important risk factor for AL. This is closely related to the anatomical characteristics of low rectal cancer. The low rectum is deep in the pelvis, with limited space. The anastomosis site is close to the anal sphincter, making surgical manipulation difficult and anastomotic tension higher. Also, the blood supply to the low rectum is relatively weak, and it faces more mechanical stimulation from stool passage. These factors together lead to a significantly higher incidence of AL after low rectal cancer surgery compared to high rectal cancer. Fukada et al. (19) found that tumor distance less than 6cm from the anal verge significantly increased the incidence of AL. Zheng et al. (20) also identified tumor distance from the anus as a key factor for AL.

Longer operation time and high BMI were confirmed as the strongest risk factors. Longer operation time was the predictor with the largest contribution in the model. This highlights the critical impact of surgical operation duration on postoperative recovery. Prolonged surgery leads to longer exposure of abdominal tissues to air, increasing infection risk (21). Also, longer duration of surgical trauma enhances the body’s stress response, releasing more inflammatory factors that inhibit the proliferation and differentiation of cells involved in anastomotic healing. Moreover, longer operation time is often related to complex tumor location or severe adhesions. These factors themselves increase the difficulty of handling the anastomosis. Wen et al.’s analysis (22) also supports that longer operation time increases risk. Obese patients have abundant intra-abdominal fat tissue. This increases surgical difficulty, leading to poor exposure of the anastomotic site and higher anastomotic tension, affecting blood supply. Also, fat tissue itself has pro-inflammatory properties. It can trigger systemic chronic inflammation by secreting inflammatory factors, inhibiting tissue repair ability, thereby increasing the risk of AL (23).

This study found that lower blood loss was an independent risk factor for anastomotic leakage. This paradoxical finding may be explained by the fact that extremely low blood loss often occurs in scenarios where surgical dissection is excessively meticulous or energy devices such as ultrasonic scalpels are extensively used. While reducing visible blood loss, such maneuvers may cause cumulative thermal damage and microcirculatory disruption, thereby compromising anastomotic blood supply. Furthermore, in low rectal cancer surgery, excessive traction or electrocautery in pursuit of a “bloodless surgical field” may damage the mesorectal vascular arcade, leading to ischemia of the distal bowel. Therefore, within the range of routine blood loss observed in this study (mean approximately 110 mL), relatively higher blood loss might be an indirect indicator of well-perfused and viable tissue. However, we acknowledge that this inverse association may also partly reflect residual confounding, and that lower blood loss could serve as a surrogate marker of greater surgical difficulty rather than a direct causal factor. Of note, key variables not included in our analysis—such as the duration and intensity of energy device use, and the surgeon’s hemostatic technique and experience in laparoscopic procedures—may influence both blood loss and anastomotic healing. Future studies incorporating detailed intraoperative data are warranted to elucidate these associations.

It is particularly noteworthy that this study first revealed the independent predictive value of the Systemic Inflammation Response Index (SIRI) for AL after rectal cancer surgery. The prediction model clearly supports the key role of SIRI. SIRI integrates neutrophil, lymphocyte, and monocyte counts. It can more comprehensively reflect the host’s systemic inflammatory state. Its elevation suggests a pro-inflammatory and immunosuppressive state characterized by increased neutrophils and decreased lymphocytes (24). Neutrophils and monocytes can release large amounts of inflammatory factors, triggering local inflammation. Inflammation disrupts the tissue repair microenvironment and inhibits fibroblast proliferation and collagen deposition. Also, neutrophils can secrete factors like matrix metalloproteinases, affecting the balance of collagen synthesis and degradation (25). Besides, several studies have revealed the role of lymphocytes in rectal cancer. Lymphocytes can eliminate tumor cells and other microbes, playing a role in tumor immunity (26), In the process of AL, immunosuppression caused by lymphocytopenia weakens the clearance of pathogens and cell proliferation/migration during tissue repair, possibly leading to AL.

In addition, the prediction model constructed in this study holds greater oncological significance, which merits in-depth exploration. Multiple previous studies have confirmed that AL following colorectal cancer surgery is closely associated with adverse outcomes, including increased local recurrence rates and reduced long-term survival (27, 28). A plausible mechanism is that AL can induce local inflammation and a systemic inflammatory response, and the released proinflammatory mediators may trigger local interstitial hyperplasia and confer drug and chemotherapy resistance in tumor cells (29, 30). Therefore, the value of identifying patients at high risk of AL extends beyond reducing the incidence of short-term complications, and may even exert a significant impact on oncological prognosis. In this context, the nomogram established in this study can serve not only as a tool for assessing surgical safety, but also as an auxiliary tool for clinical oncological decision-making. For patients identified as high-risk for AL, clinicians may consider appropriately relaxing the indications for prophylactic ileostomy, more cautiously planning the interval between chemotherapy and surgery, or enhancing postoperative monitoring to detect AL and potential signs of tumor recurrence at an early stage.

In recent years, several prediction models for AL have been developed. But most rely on demographic, oncological, and surgery-related variables. Although some studies recognize the importance of inflammation, they are mostly limited to single indicators like C-reactive protein or Neutrophil-to-Lymphocyte Ratio (NLR) (31–33). The core innovation of this study is the systematic evaluation and confirmation of the superior predictive value of composite inflammatory indicators represented by SIRI. Through strict screening by LASSO regression, SIRI stood out among many inflammatory indicators. Traditional indicators like NLR were not included in the final model. This suggests that in the prediction model of this study, SIRI might be a more discriminative quantitative tool for systemic inflammatory state than traditional single inflammatory indicators.

The present findings should be interpreted within the evolving framework of precision oncology, in which clinically accessible biomarkers are increasingly combined with molecular, immune-related, and imaging-derived parameters to refine patient stratification. In colorectal cancer, host-related biological variability has already been shown to influence treatment outcomes, as exemplified by the association between FcγRIIIa polymorphisms and response to anti-EGFR monoclonal antibodies in metastatic disease (34). Similarly, radiomics approaches based on preoperative EOB-MRI have demonstrated the potential to predict relevant pathological and clinical outcomes following liver resection for colorectal liver metastases, thereby supporting the role of non-invasive biomarkers in personalised surgical oncology (35). In this context, the incorporation of SIRI into a preoperative nomogram may represent a pragmatic extension of this paradigm: rather than relying exclusively on anatomical or technical variables, perioperative risk assessment may benefit from integrating systemic inflammatory status as a surrogate of host-tumour interaction, tissue repair capacity, and susceptibility to postoperative complications.

It is also important to consider the clinical consequences of false-negative predictions. Patients who are incorrectly classified as low-risk by the nomogram but subsequently develop AL may not receive prophylactic stoma, may undergo less intensive postoperative monitoring, and may experience delayed diagnosis and intervention, with potentially serious surgical and oncological consequences. Therefore, this nomogram should be regarded as a supplementary tool to inform perioperative decision-making rather than as a standalone determinant. Decisions regarding prophylactic stoma and postoperative management should integrate the model’s predictions with intraoperative findings and the surgeon’s clinical judgment.

This study has several limitations. First, it is a retrospective, single-center study, and the validation set was derived from an internal split of the same cohort rather than an independent external population. Therefore, the model’s generalizability to other centers or patient populations remains unproven, and the observed AUC values may partly reflect single-center homogeneity. Second, as a retrospective study, although we controlled for multiple confounding factors, there may still be unmeasured confounding bias. Additionally, detailed intraoperative variables specific to laparoscopic surgery, such as the duration and intensity of energy device use and surgeon technique, were not recorded, which may have introduced additional bias, particularly regarding the interpretation of the observed inverse relationship between blood loss and anastomotic leakage. Third, patients who received neoadjuvant chemoradiotherapy were excluded from this study. While this exclusion strategy ensured cohort homogeneity and avoided the confounding effects of radiation-induced tissue changes on inflammatory markers, it also means that the constructed nomogram is not applicable to patients who have undergone neoadjuvant chemoradiotherapy, a population that constitutes a substantial proportion of real-world rectal cancer patients. Therefore, dedicated prediction models are needed for this subgroup in the future. Future research will involve multicenter studies with larger sample sizes covering different regions to further validate the clinical utility of this model and improve its external generalizability.

## 5 Conclusion

This study developed and preliminarily validated a nomogram that integrates preoperative systemic inflammation response index (SIRI) with essential clinical characteristics. The model provides good predictive accuracy for individualized risk assessment of anastomotic leakage following anterior resection for rectal cancer and may serve as a complementary tool to inform perioperative decision-making. Despite the inherent limitations of a retrospective study, this model provides clinicians with an intuitive and practical preoperative risk assessment tool. It helps achieve early identification and stratified management of high-risk patients. It has positive clinical significance for optimizing perioperative decision-making and improving patient prognosis.

## Data Availability

The raw data supporting the conclusions of this article will be made available by the authors, without undue reservation.
